# The "Tracked Roaming Transect" and distance sampling methods increase the efficiency of underwater visual censuses

**DOI:** 10.1371/journal.pone.0190990

**Published:** 2018-01-11

**Authors:** Alejo J. Irigoyen, Irene Rojo, Antonio Calò, Gastón Trobbiani, Noela Sánchez-Carnero, José A. García-Charton

**Affiliations:** 1 Centro para el Estudio de Sistemas Marinos (CESIMAR), Consejo Nacional de Investigaciones Científicas y Técnicas (CCT CENPAT–CONICET). Boulevard Brown, Puerto Madryn, Chubut, Argentina; 2 Depto. Ecología e Hidrología–Universidad de Murcia, Campus de Espinardo, Murcia, Spain; 3 Université Côte d’Azur, CNRS, FRE 3729 ECOMERS, Parc Valrose, Nice, France; Leibniz Centre for Tropical Marine Research, GERMANY

## Abstract

Underwater visual census (UVC) is the most common approach for estimating diversity, abundance and size of reef fishes in shallow and clear waters. Abundance estimation through UVC is particularly problematic in species occurring at low densities and/or highly aggregated because of their high variability at both spatial and temporal scales. The statistical power of experiments involving UVC techniques may be increased by augmenting the number of replicates or the area surveyed. In this work we present and test the efficiency of an UVC method based on diver towed GPS, the Tracked Roaming Transect (TRT), designed to maximize transect length (and thus the surveyed area) with respect to diving time invested in monitoring, as compared to Conventional Strip Transects (CST). Additionally, we analyze the effect of increasing transect width and length on the precision of density estimates by comparing TRT vs. CST methods using different fixed widths of 6 and 20 m (FW3 and FW10, respectively) and the Distance Sampling (DS) method, in which perpendicular distance of each fish or group of fishes to the transect line is estimated by divers up to 20 m from the transect line. The TRT was 74% more time and cost efficient than the CST (all transect widths considered together) and, for a given time, the use of TRT and/or increasing the transect width increased the precision of density estimates. In addition, since with the DS method distances of fishes to the transect line have to be estimated, and not measured directly as in terrestrial environments, errors in estimations of perpendicular distances can seriously affect DS density estimations. To assess the occurrence of distance estimation errors and their dependence on the observer’s experience, a field experiment using wooden fish models was performed. We tested the precision and accuracy of density estimators based on fixed widths and the DS method. The accuracy of the estimates was measured comparing the actual total abundance with those estimated by divers using FW3, FW10, and DS estimators. Density estimates differed by 13% (range 0.1–31%) from the actual values (average = 13.09%; median = 14.16%). Based on our results we encourage the use of the Tracked Roaming Transect with Distance Sampling (TRT+DS) method for improving density estimates of species occurring at low densities and/or highly aggregated, as well as for exploratory rapid-assessment surveys in which divers could gather spatial ecological and ecosystem information on large areas during UVC.

## Introduction

A variety of methods has been used to estimate reef fish diversity, abundance and size; sampling methods include both capture techniques such as ichthyocides, nets and traps, and observational (non-destructive) methods such as video recording and underwater visual censuses (UVC) [1–5]. UVC techniques are the most commonly used method in shallow and clear waters worldwide [6–10]. Beyond being non-lethal, thus appropriate to be used in marine protected areas (MPAs) and for long-lived, rare and/or threatened species, UVC techniques are suitable for a wide range of fish sizes and behaviours as well as habitat types (especially in architecturally complex ones), and, importantly, they are easy to learn by divers, who may simultaneously and rapidly register information on environmental variables and fish behaviour [5,11]; for these reasons, UVC techniques are extremely flexible for any sampling design to be implemented in the field, so that a great variety of ecological questions can be dealt with [7,12]. Drawbacks of UVC techniques, however, also exist, such as physiological constraints of SCUBA diving (especially in deep, cold, strong current and/or turbid conditions), individual response of fishes (either attraction or escape), and bias in the estimates of abundance of cryptic, elusive, hidden or/and small-sized fishes [7, 12–16]. Consequently, a series of methodological studies dealing with these potential biases have been carried out in the last decades [5, 11, 15, 17–19].

Standard UVC techniques can be classified on the basis of the shape of the sampling unit, typically distinguishing among (1) strip transects [20], in which the diver records all fish detected along a path of fixed width and length; (2) roaming transects [21], in which the diver records all fish detected along a free path (or following a given isobath) of fixed width during a prefixed time; and (3) stationary point counts [22], in which the diver records all fish detected within a circular area of fixed radius. Strip transects are currently adopted in most ichthyofauna monitoring programs because they are better suited for a wide range of environments and types of reef fish assemblages [23,24].

Most UVC methods were conceived to efficiently gather information on fish assemblages that can be composed by a wide spectrum of species, sizes, behaviours and densities [12, 25, 26]. However, the selection of a proper UVC technique is particularly problematic in species occurring at low densities and/or highly aggregated (i.e., schooling species, such as damselfishes or bogues), or patchily distributed species (such as groupers) [27, 28] due to the high variability of their density at both spatial and temporal scales [11, 13, 29, 30, 31, 32]. Among these scarce, patchy and/or highly aggregated species, charismatic and commercially important reef fishes are often found, generally targeted by monitoring programs because of their relevance in fisheries management, conservation and MPA effectiveness analysis [33, 34]. Regarding the spatial perspective, the patchy distribution of some species increases the variability between replicates, while species occurring at low densities could not be detected [27]. On the other hand, as fishes are mobile animals, estimations vary at different temporal scales (minutes, days, weeks), and furthermore, the magnitude of this variability increases systematically as the mean of abundances decreases [11, 30]. Consequently, determining population changes on this species group might be difficult without having a large number of replicates.

The statistical power of UVC-based studies could be increased by augmenting either the number of replicates, the area surveyed [11, 30, 35] or using the distance sampling method, which considers intra-transect variance that is absent in other methods [35]. The number of replicates is usually constrained by sampling costs, logistics or/and dive time. In the case of strip transects, census area is determined by the length and width of the sampling units. Transect length is determined by a trade-off between time consumed for each replicate and the maximum possible length (which, in turn, is constrained by tape length, reef size, habitat heterogeneity, diving time, etc.). [7, 12, 19, 36]. Additionally, long transects could be constrained by habitat heterogeneity or by the size of the study site in the case of very long transects. Transect width, which could be limited by visibility constraints (particularly in temperate environments),is susceptible to optimization analysis [37]. Furthermore, it is noteworthy that both dimensions of the transect are subject to methodological considerations which may influence the results, as is the case of the reported impact of transect’s dimensions in diversity indices and the first-and-end transect section effect on fish abundance estimations [19, 36]. In some cases, the area surveyed can be maximized by performing roaming censuses [21], in which the diver swims in a free direction or following a predetermined direction. These surveys usually have a prefixed time, hence sighting frequency rather than fish density is quantified [21] since the method does not account for the area covered by the survey. This limitation is probably one of the main reasons of its limited use in studies involving UVC. From this perspective, the roaming transect could be refined by tracking the transect path and obtaining an accurate estimation of the area covered by the survey [27, 38].

Otherwise, transect-based samplings (usually terrestrial) take advantage of the Distance Sampling method (DS), a methodology that allows estimating the density of animals, plants or any event of interest taking into account large width census areas [39, 40]. Underwater, the DS method is a technique in which the observation/count distance could be increased up to the visibility limits since the distance of the observed individual from the transect (in transect-based sampling) or the observation centre (in stationary point count sampling) is used to estimate densities [39–41], thus avoiding the constraints of limiting the survey to a given width or radius. DS is based on the probability of detection of each observed individual, which can be estimated from the frequency distribution of the recorded sighting distances [39, 42, 43]. Detection probability generally decreases with increasing distance from the observer, and the shape of the sightings distribution is highly influenced by fish size, wariness and crypticity [42, 44]. Besides their usefulness for integrating information from large census areas, distances may provide further information on the behaviour of animals, which is extremely relevant in comparative studies among sites with different levels of human disturbance (i.e. MPA effectiveness evaluation studies) [16, 45, 46]. For example, in areas open to fishing, individuals of commercial species may keep themselves away from divers at distances longer than the transect width, while in areas with fishing restrictions they could show no diver avoidance [44, 45]. From this perspective, opposite to the case of terrestrial ecology, the use of DS to address species behavioural issues is concentrated in studies and monitoring programs carried out in the eastern Pacific Ocean [35, 42, 43, 45].

Here we describe and test the efficiency (estimated as its capacity to maximize the area surveyed) and precision (estimated as the coefficients of variations of density estimations) of the Tracked Roaming Transect (TRT), an UVC method designed to maximize transect length (and thus the surveyed area) with respect to diving time invested in monitoring. The method was specifically designed to survey commercial and charismatic, medium- and large-size fish species commonly found at low densities (such as groupers or sharks) and/or threatened species [28, 31, 32]. Furthermore, we analyzed the effect of increasing transect width on the precision of abundance estimations by comparing different fixed transect widths and the Distance Sampling (DS) which could maximize the transect width up to the visibility limits. Additionally, since with the DS method distances of fishes to the transect line have to be estimated, and not measured directly as in terrestrial environments [46], errors in estimations of perpendicular distances can seriously affect DS density estimations as well as estimations based on fixed belt transects [17, 19]. To assess the occurrence of these errors and their dependence on the experience of the observers performing fish UVC, a field experiment using wooden fish models was performed to test the precision and accuracy of density estimations with DS method as well as with fixed transect widths used.

## Methods

### Species and study sites

**Fig 1:**
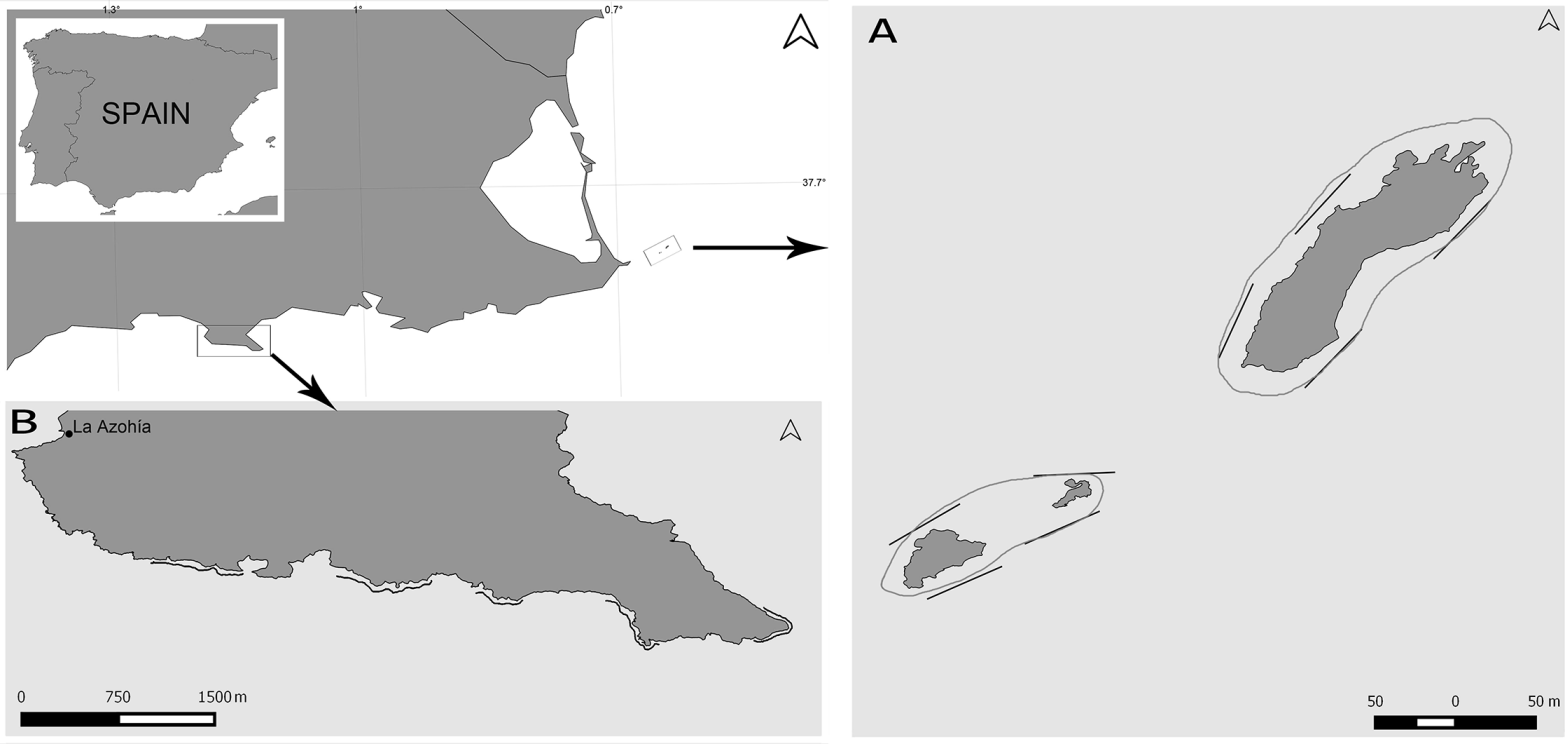
Study sites. (A) Islas Hormigas and Hormigon in Cabo de Palos Marine Reserve (37.6550° N -0.6497° E). The distance between islands is not to scale to ease the representation. (B) Cabo Tiñoso (37.5370° N -1.1419° E). Straight lines: 50-m Conventional Strip Transects. Curved lines: Tracks of the Tracked Roaming Transects.

This study was carried out in July 2014 in Cabo de Palos—Islas Hormigas marine reserve (hereafter CP), and in Cabo Tiñoso (CT), a neighbouring unprotected area at the time of sampling (it is protected since July 2016), both locations situated along the coast of Murcia (SE Spain, Mediterranean Sea) (Fig 1). The marine reserve of CP was established in 1995 under fisheries legislation, and it occupies 1931 ha, from which 270 ha form the core no-take area (centred in 37°39'21"N, 0°38'57" W, around the Hormigas islands), where this study was undertaken. For its part, CT is the projection into the sea of a coastal cliff that extends for ~7 km (Fig 1).The two locations are characterized by clear water (visibility > 20 m most of the year in normal conditions) and long sections of steep reefs and large rocky boulders down to ~ 40 m depth.

Six species were selected to be surveyed in this study, three grouper species (Epinephilidae) [dusky grouper *Epinephelus marginatus*, goldblotch grouper *Epinephelus costae*, and mottled grouper *Mycteroperca rubra*], common dentex (*Dentex dentex*, Sparidae), brown meagre (*Sciaena umbra*, Sciaenidae) and common eagle ray (*Mylobatis aquila*, Myliobatidae). All these species are medium-to-large sized, nekton-benthic fishes with similar habitat requirements and together compose part of the ichthyofauna of coastal rocky habitats [7, 12, 25]. These species are among the most important for commercial and recreational fisheries [47] and among the most charismatic for SCUBA divers in the Western Mediterranean Sea [48]. They are also among the most favoured by protection measures within MPAs in the Mediterranean Sea [49].

### Comparison of underwater visual census methods

Two types of transects were performed: the Conventional Strip Transects (CST), in which the transect length is marked with a 50-m fiberglass tape measure [25] and the Tracked Roaming Transects (TRT) method, for which transects of variable length were performed (see below for further explanation). For both CST and TRT methods, visual counts were done in such a way that three census widths of 6, 20 and 40 m (3, 10 and 20 m each side of the transect, respectively) could be considered simultaneously in the same dive/sampling operation (see below). While the 6- and 20-m stretches were of fixed widths (called FW3 and FW10, respectively), density within the 40-m width was computed using a Distance Sampling (DS) methodology, so that six census methods (called CST-FW3, CST-FW10, CST-DS, TRT-FW3, TRT-FW10, and TRT-DS respectively) were compared. It is noteworthy that using *a posteriori* recorded distances it is likely that abundance estimates differ from yields resulting from fixed widths considered from the start. However, since this work is not focused on the comparisons of abundance estimations, this possible bias are not relevant.

### TRT method

**Fig 2:**
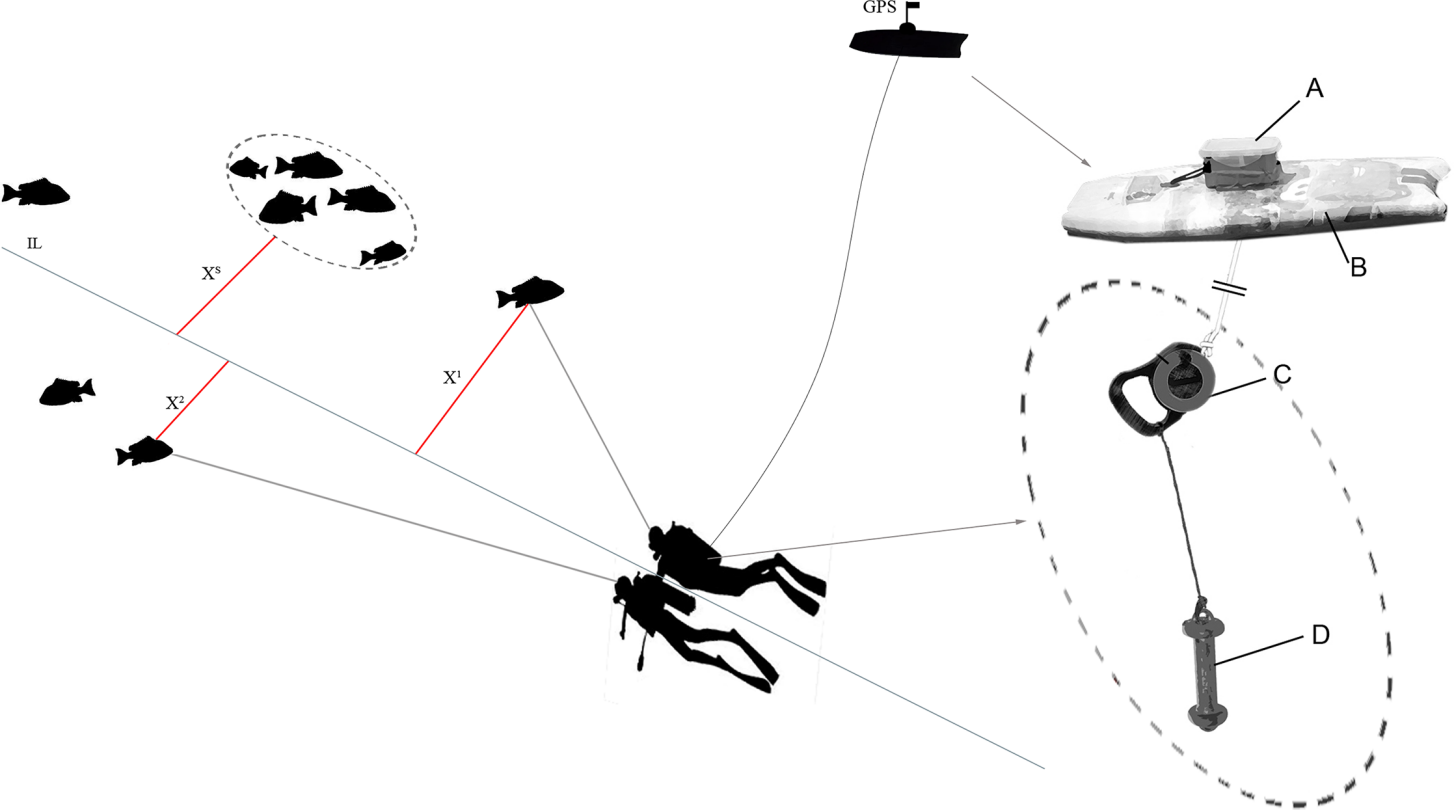
Scheme of the count method using the Distance Sampling method. IL = imaginary line. X^1-2^ = perpendicular distances of solitary individuals to the transect line. X^3^ = perpendicular distance of a school of fishes to the perpendicular line. The equipment (Fig 2) is illustrated above divers, like at the surface. Inset: The Tracked Roaming Transect equipment. A = Waterproof case for GPS. B = Body Board Slate. C = Diving reel. D = Weight.

The TRT method proposed here is based on a GPS density survey designed to asses reef fish spawning aggregations [38] and other experiences of diver towed GPS techniques [50, 51, 28]. With this technique it is possible to measure the distance covered by geo-referencing start/end census points (along with the track recorded by the GPS) or to geo-reference any object, event or spatial feature of interest. The equipment, constructed *ad hoc* for this study, consists of a GPS mounted on a body-board, a diving reel with a monofilament of 1 mm diameter and a 3 kg weight (Fig 2). The reel is tied 0.8 m above the weight, thus the monofilament length can be regulated easily by the diver (Fig 2). The diver regulates the line length before starting the census so that the weight hangs from the board. Then the diver drags the equipment pulling on the line and thus the weight maintains the board vertically over the diver, minimizing the displacement of the board from the diver position. Photographs, which record the time or the day time registered during the dive, are used to geo-locate points or events (transect start/end point, fish counts, habitat features, etc.). The only requirement is the exact correspondence between camera or diving computer and GPS clocks or to take a picture of the GPS screen with the underwater camera before or after the dive to calculate the time difference between devices. Then, using a geographic information system (GIS) software, the time of the start/end point is associated with a waypoint of the track recorded by the synchronized GPS. The resulting track between start and end points is equal to the UVC transect length (for this study the GPS was set to construct the track, loading a waypoint every 3 seconds). Additionally, using free software for geo-referencing of photographs (in our case: http://jriguera.github.io/photoplace) it is possible to match the GPS data timestamps with the digital image timestamps, extracted from their embedded EXIF tags, and create a geospatial data set.

### Procedure of visual census sampling operation

For both CST and TRT methods, divers conducted censuses in pairs as follows: once the observers defined the starting point of the census, they followed an imaginary line at a constant average depth of 16 m. Each left or right diver was responsible for counting fishes present only along his/her side of the sampling path, i.e. the left and right half of the transect, respectively. The two divers swam ahead simultaneously and continuously, while one of them unrolled the measuring tape (in the case of CST) or pulled GPS positioned at the surface above their vertical axis (in the case of TRT).

In all cases, divers recorded individuals of the selected fish species within 20 m from their side of the imaginary line. Divers estimated the first-sight perpendicular distance (to the nearest meter) between each observed individual fish encountered and the imaginary line (Fig 2). For groups of fish of the same species (schools), the distance from the closest fish of the group (Fig 2) was recorded and then the number of individuals was estimated. This information is necessary for the use of DS density estimations methods (see below). Distances were noted in 1-m intervals and fish observed at estimated distances beyond 20 m were not recorded. Fish size was recorded in 10-cm classes, and only fishes larger than 10 cm were included in the census in order to avoid possible biases associated with small size categories [13]. When fish moved from one side of the transect to the other, this was registered, so that, after the census, both divers immediately cross-checked this information in order to avoid a double count of individuals.

### Sampling design and data analysis

In CP, 4 TRT following the 16-m isobath were conducted encircling each of the two islands of the Hormigas archipelago in the no-take zone (Fig 2), with one transect situated on the windward side and another one in the leeward side of each island. Eight CST 50-m long were randomly located on the leeward and windward side of the two islands also following the 16-m isobath (Fig 2). The number of CSTs was determined in order to spend a diving time comparable to the time invested for TRT. In CT, 5 TRT were conducted, but in this case, due to the extremely low abundances of the studied species in this location, CST was disregarded. Instead, CST-like transects were simulated taking advantage of the fact that observations of each individual or school during TRT were geo-located and their distance to the transect line was noted. With this recorded spatial distribution of fishes, a Monte Carlo simulation was conducted to reproduce 19 random 50-m CST laid over the TRT tracks, avoiding overlaps among them; this number is the time-equivalent number of CST in relation to the TRT sampling effort. For each simulation, fishes located within the 19 CST tracks were counted as "detected fish". Detection within 3 and 10 meters from transect were used to calculate the FW3 and FW10 estimators, respectively. Mean and standard deviation of the number of fishes detected in the CST were computed for each simulation (among the 19 CST) for each species. A total of 10^5^ simulations were computed and global mean and the coefficient of variation among them were calculated for each species.

Comparisons of the six census methods were focused on the efficiency (calculated as the area covered per unit time) and precision (estimated as the coefficients of variation) of abundance estimates. In all cases, densities (D) were referred to as the number of fish per 300 m^2^ (which is equivalent to a CST-FW3 transect), and the coefficient of variation (CV) was considered for each location and combination of transect method as a standardized estimator of the variability. The CV was derived from the square root of the variance (i.e. standard error) divided by the density estimate. Densities and CV in DS method were calculated using the free software DISTANCE [39, 40]. Unlike the methods mentioned before in which variance is derived from differences in the number of individuals recorded per unit of area among transects, the sources of uncertainty in DS estimates involve three components that are combined additively to compute the CV: the empirical variability in the encounter rate of clusters; the variability in cluster size; and the uncertainty due to the maximum likelihood estimation of the detection-function parameters [39, 40]. Accuracy in this section was not analysed since true density and fish distribution among different census areas comprised in this study were unknown.

### Accuracy of abundance estimations: A field experiment

In order to simulate a situation with fishes situated at known distances from the census line, 18 light brown wooden fish of 40 cm in length were fixed along a 50-m transect line placed at 10 m depth on a rocky reef within the CP marine reserve. The wooden fish were suspended between 0.3 and 0.7 m over the bottom and placed at random distances between 0 to 20 m on the right side of a transect line by a pair of divers who did not participate in the experiment.

The location, position above the bottom and colour of wooden fish were chosen in order to simulate an equivalent detection probability of real fishes, including those situated behind rocks in the census area. Each diver had to estimate independently the distance of each wooden fish perpendicular to the transect line while swimming along the transect at a constant speed, this procedure being repeated 3 times by the set of divers, with the wooden fish placed at different positions each time (thus, N = 54, i.e. 18 per transect, for each diver). During data analysis, the three passes were considered as a unique transect of 150 m in length. Divers had no previous information on the number of wooden fish present in the transect nor the distance range of the fish from the transect line. This procedure was replicated in two different occasions. On a first occasion, five divers performed the experiment: three experienced divers (with > 10 years of experience in visual census) and two non-experienced divers (with no previous experience in UVC). One of the non-experienced divers had a vast experience as scuba diver and the other had much less (< 20 dives). On a second occasion, three divers, one of them an experienced diver and the other two non-experienced, performed the experiment. In this second experiment, wooden fish were numbered and divers annotated the number of each fish, so that real versus estimated distance could be compared. The accuracy of the estimates of each occasion was measured comparing actual total abundance with those estimated from each diver’s observations using FW3, FW10, and DS estimators.

## Results

### Comparison among census methods

Nine TRTs were performed in both studied locations (CP and CT) covering a distance of 4829 m for a total of 255 minutes of diving time per pair of divers (Fig 1): 4 TRTs were performed in CP (covering 1221 m in 69 min) and 5 TRTs in CT (3608 m in 186 min). In CP, 8 CSTs were performed in four dives, requiring a total of 90 min of diving time per pair of divers. In CP, the ratio between the distance covered in an CST compared with a TRT (distance in meters covered on CST per minute of diving time / distance covered on TRT per minute of diving time) was 0.26 (i.e.: the TRT method was 4 times more efficient than CST). A total of 1266 individuals of the 6 selected fish species were counted in CP, 827 by the 9 TRTs and the remaining 439 fishes by the 8 CSTs. Overall, 93.8% of the fish were counted in CP, despite the sampling effort (in terms of distance surveyed) in this site being 52% lower than in CT. Groupers (*E*. *marginatus*, *E*. *costae* and *M*. *rubra)* and *M*. *aquila* were observed mostly solitary or in isolated pairs. On the other hand, *S*. *umbra* was mostly recorded in schools (75% of detections–up to 80 individuals per detection) and *D*. *dentex* was recorded in schools up to 30 individuals in 25% of cases.

In order to make valid estimations for distance distribution, at least ~30 observations are necessary [39, 40]. For this reason, density estimates following the DS theory were calculated only for *E*. *marginatus* and *D*. *dentex*, and the total number of groupers (i.e. considering the three species jointly).

**Fig 3:**
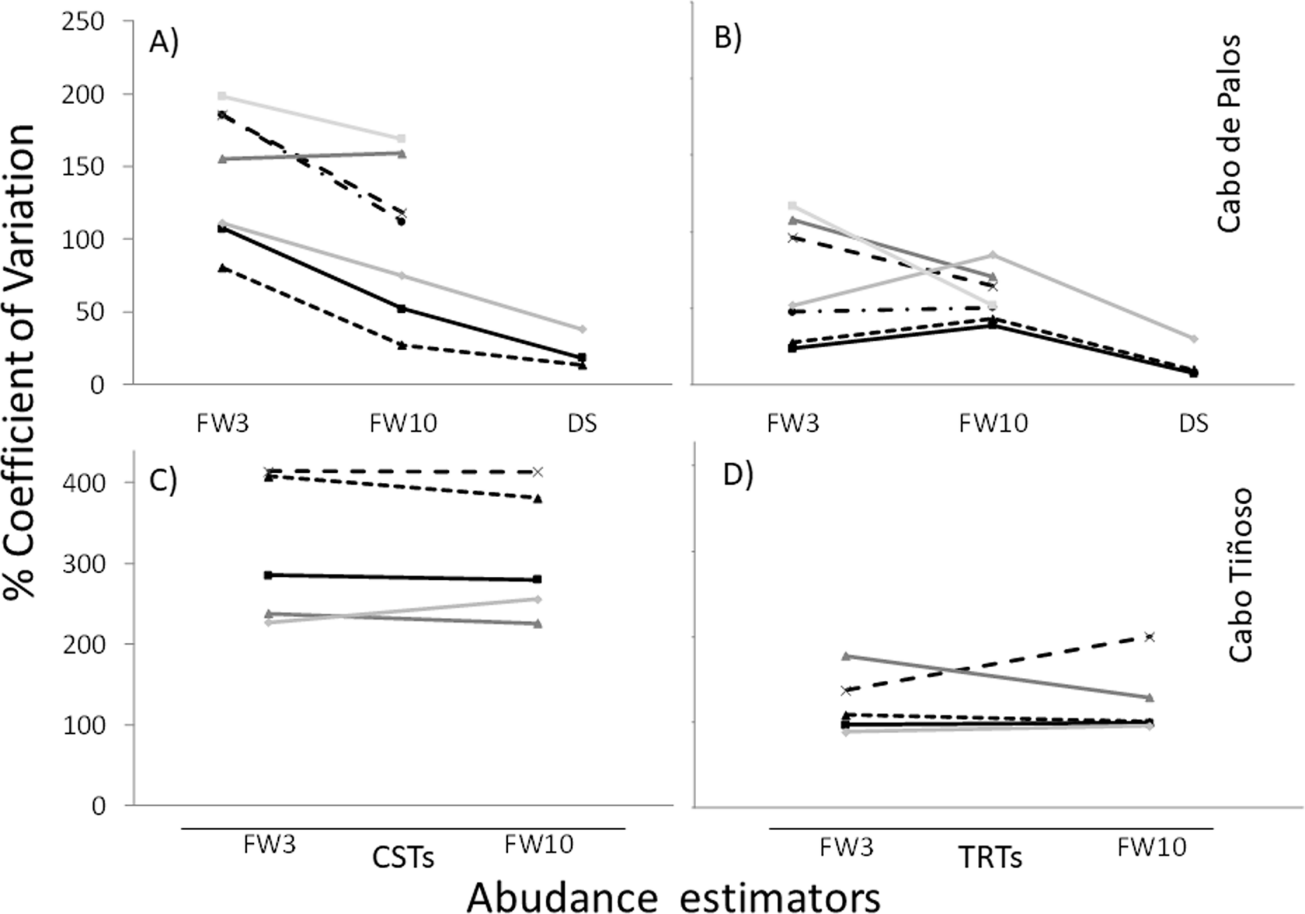
Coefficients of variation in percentage of density estimations of the different methods combinations in Cabo de Palos and Cabo Tiñoso. (A) Conventional Strip Transects in Cabo de Palos. (B) Tracked Roaming Transects in Cabo de Palos. (C) Conventional Strip Transects in Cabo Tiñoso (data obtained from simulations). (D) Tracked Roaming Transects in Cabo Tiñoso. Black solid lines with square markers: total groupers. Short dashed line with triangle markers: *Ephinepelus marginatus*. Long dashed line with x marker: *Ephinepelus costae*. Dotted and dashed line with circle markers: *Mycteroperca rubra*. Grey solid lines with triangle marker: *Sciena umbra*. Soft grey solid lines with diamond marker: *Dentex dentex*. Ligth grey solid line with square marker: *Myliobatis aquila*. DS: Distance Sampling. FW3: Fixed width of 3 m for each side of the transect. FW10: Fixed width of 10 m for each side of the transect.

The coefficients of variation (CVs) of estimations based on TRT counts were smaller than those based on CST (Table 1; Fig 3). When CVs are compared between transects methods, CVs attain on average 57.3, 42.4 and 47.7% for the FW3, FW10 and DS estimators, respectively, considering both sampling locations (Table 1; Fig 3).

**Table 1:** Density estimations (D) (fish/300m^2^) and coefficients of variations (CV) for studied species and the total of groupers for different combinations of transects types and census widths in Cabo de Palos and Cabo Tiñoso. Note that CST values in Cabo Tiñoso were derived from simulations. CST = Conventional Strip Transect. TRT = Tracked Roaming Transect. FW3 and FW10 = Fixed Width of 3 and 10 m for each side of the transects, respectively. DS = Distance Sampling Method. The census area of each survey is detailed in m^2^ considering total transect lengths and 6 and 20 m width for FW´s estimators and 40 m width for the DS estimator.

Site	Transect type/ Census method/ Area (m^2^)	Total groupers		*E*. *marginatus*		*M*. *rubra*		*E*.*costae*		*S*. *umbra*		*D*. *dentex*		*M*. *aguila*	
		D	%cv	D	%cv		%cv	D	%cv	*D*	%cv	*D*	%cv	*D*	%cv
Cabo Palos	CST FW3 2400 m^2^	3.8	107.4	2.0	80.2	1.3	185.2	0.5	185.2	0.8	155.3	1.3	111.1	0.4	198.4
	TRT FW3 7400 m^2^	4.4	23.6	3.0	27.3	1.0	47.3	0.3	96.0	6.8	107.8	0.6	51.5	0.1	116.7
	CST FW10 8000 m^2^	3.3	52.0	2.1	27.0	1.0	112.0	0.2	118.0	12.2	159.0	0.6	75.0	0.2	169.0
	TRT FW10 24666 m^2^	1.9	38.7	1.5	42.8	0.3	50.3	0.1	64.2	3.6	70.5	1.4	84.7	0.1	51.6
	CST DS 16000 m^2^	4.2	18.1	3.6	13.5	-	-	-	-	-	-	1.5	38.0	-	-
	TRT DS 49000 m^2^	2.9	7.4	2.6	9.6	-	-	-	-	-	-	1.2	30.0	-	-
Cabo Tiñoso	CST FW3 5700 m^2^	0.1	285.0	0.1	408.0	-	-	0.1	414.0	0.2	238.0	0.3	227.0	-	-
	TRT FW3 21600 m^2^	0.1	96.9	0.1	108.3	-	-	0.0	137.3	0.2	177.3	0.2	88.7	-	-
	CST FW10 19000 m^2^	0.1	279.6	0.1	381.0	-	-	0.1	413.6	0.4	225.6	0.5	255.6	-	-
	TRT FW10 72000 m^2^	0.1	99.0	0.0	100.2	-	-	0.0	200.0	0.1	128.6	0.1	95.9	-	-

On the other hand, within counts on CSTs and TRTs, values of CV tended to diminish as the abundance estimators incorporated wider census areas in Cabo de Palos (with the exception of three species using TRT-FW10 method) and remained of similar magnitude for counts performed in Cabo Tiñoso (Table 1; Fig 3).

### Accuracy of abundance estimations—Field experiment

Non-experienced divers systematically underestimated perpendicular distances (mean distance estimated = 5.2 m; actual mean distance = 8.7 m), and consequently overestimated abundance values (average = 64%) for the three estimators calculated [39] (Tables 2 and 3). Without considering the estimations made by non-experienced divers, abundance estimations of wooden fish using the FW3, FW10 and DS estimators, merging data from the two experimental occasions, ranged between 0.1 to 31% of difference compared to actual abundances (average = 13.1%; median = 14.2%). On the second occasion, in which the actual distance from each wooden fish was known, the average distance estimation error was 19.2% ± 19.86 standard error. Regarding the DS technique, the abundance estimations of the four experienced and three of the non-experienced divers were similar to the actual abundance of wooden fish (number of wooden fish = 54 and density = 0.0180 individuals m^2^) (Table 2), thus the actual abundance was within the confidence intervals estimated with count data from these seven divers. The number of wooden fish estimated ranged between 53 and 70, and the coefficients of variation (CV) of the estimations ranged between 13.1 and 17%. The number of wooden fish observed by each diver ranged between 39 and 46 out of the 54 wooden fish, with no trend observed between the number of fish detections per diver and the accuracy of abundance estimation. The actual mean of perpendicular distances of wooden fish to the transect line was 8.7 m in both experiments, and the mean distances estimated by the divers ranged between 7.7 and 9.6 m (Table 2).

**Table 2:** Number (N) of wooden fishes estimated with the Distance Sampling (DS) method by the experienced and non-experienced divers for the total census area (3000 m^2^) and the confidence interval (N CI 95%) at p < 0.05. DS estimator: the best fit from the nine estimators tested with the DISTANCE software. D = Density per 300 m2. %CV = Coefficient of variation of the estimation in percentage. N detected (%) = Number of wooden fishes detected by divers and the percentage of the total wooden fish. MDD = Mean distance detection derived from visual estimations and standard deviation (SD) by divers. "*" Actual number of wooden fishes. "**" Actual mean distance.

Diver		DS estimator	N*	N CI 95%	D (300 m^2^)	%CV	N detected (%)	MDD**(SD)
Experienced	1	Half-normal/Cosine	53	39–73	5.3	15.9	46 (85)	9.6 (5.5)
	2	Half-normal/Cosine	60	45–80	6.0	14.1	40 (74)	8.1 (5.1)
	3	Uniform/Cosine	53	39–77	5.3	15.9	39 (72)	9.2 (6.2)
	4	Half-normal/Cosine	56	39–79	5.6	17.1	42 (78)	9.1 (5.7)
Non Experienced	1	Half-normal/Cosine	55	38–79	5.5	17.0	41 (75)	9.4 (5.7)
	2	Half-normal/Hermite	99	57–173	10.0	28.0	44 (81)	5.2 (3.1)
	3	Uniform/Cosine	70	54–91	7.0	13.1	44 (81)	7.7 (5.6)
	4	Half-normal/Cosine	65	48–87	6.5	14.7	44 (81)	7.9 (5.3)

* Actual N = 54

** Actual mean distance of experiments 1 and 2 = 8,7

**Table 3:** Density of wooden fishes/300 m^2^ and percentage CV estimated by the experienced and non-experienced divers using the three abundance estimators in this study (DS, FW3 and FW10). % Average estimation error: The average estimation error in percentage without considering data from one of the inexperienced divers who systematically underestimated distances.

	Diver	DS	%DS CV	FW10	%FW10 CV	FW3	%FW3 CV
Experiment 1	Experienced	5.3	15.9	5.5	12.0	4.6	50.0
		6.0	14.1	5.5	12.0	4.0	50.0
		5.3	15.9	5.1	26.0	2.3	65.0
	Non Experienced	5.5	17.0	5.1	17.0	4.0	60.0
		10.0	28.0	7.8	15.0	9.2	32.0
Actual abundances		5.4		6.7		4.6	
Experiment 2	Experienced	5.6	17.1	4.8	21.7	6.0	33.3
	Non Experienced	7.0	13.1	6.8	33.4	6.7	17.3
		6.5	14.7	6.4	37.9	7.3	31.5
Actual abundances		5.4		7.0		6.7	
**% Average estimations error**	** **	**9.9**	** **	**18.1**	** **	**9.1**	** **

## Discussion

The TRT method proposed and tested in this study increased the efficiency of UVC in comparison with CST. It thus provides new information to ecologists for selecting an UVC method to study commercial and charismatic, medium- and large-size, nektobenthic reef fish species that are commonly found at low densities. We also showed that, for a comparable time effort, variability of abundance estimations can be reduced by enlarging the transect, either by using the TRT method (i.e. increasing transect length) and/or using abundance estimators that incorporate information from larger census widths (DS or FW10) (Table 1; Fig 3). These results are in concordance with a previous DS test of UVC [19, 35, 36] and studies which suggest that increasing the census area for species occurring at low densities (provided they have high detectability) could be used as a strategy to diminish the variability of abundance estimations. However, it is important to note that it is not the case for cryptic or camouflaged species as encounter does not necessarily lead to detection [11, 13, 27, 30].

A few studies used towed GPS devices to track UVC census measuring fish densities in roaming surveys [27, 38, 52]. According to Beck et al. [27], tracking the diver’s path optimizes the sampling effort, making surveys more efficient. In this work, the high efficiency found (74% higher in TRT than CSTs and 25% higher than found by [27]) could be due to the water currents prevailing in the study area. All censuses were conducted following the direction of the water current and hence the effort and time-air consumed against the current to roll the transect tapes in CST were high with respect to diver displacements during TRT surveys. Moreover, the additional sampling area increases the chances of encountering a greater abundance of rare species and hence improves the estimates of fish diversity [27, 28]. Furthermore, the potential of the TRT method to add spatial information to species detections, such as habitat features (e.g. caves, nests), geo-localized events (reproductive or feeding aggregations), etc., gives a powerful tool to interpret patterns and processes when analyzing data [28]. In cases such as part of the study area (Cabo de Palos—Islas Hormigas marine reserve, including the Hormigas archipelago) where the sampling area (at a certain depth range) comprises all or most of the home range of fishes, serious bias related to fish movement in response to environmental (e. g. currents), or behavioural (e. g. reproductive or feeding aggregations) variables could be avoided and/or studied [53].

The density estimates obtained by using the Distance Sampling method and fixed belt transects estimators were accurate and precise for most of the divers involved in the experiments with wooden fishes. The error levels for the three estimators found in this work were of similar or lower magnitude than the lowest values of instantaneous variability reported for reef fish visual census data [11, 30]. Although several authors [15, 22, 54, 55] studied the precision of distance estimates by divers with disparate results, to our knowledge our work is the first to directly test the accuracy of density estimations following the DS theory in real field conditions. Our results encourage the use of the DS method for surveying medium- and large-sized species. However, the transect width (i.e. maximum sighting distance) probably needs to be reduced when studying small-sized species (not visible at long distances) and/or for species with fast declining detectability curves [42]. The main virtues of the DS method are the relatively low variability of estimations (which increases the power of statistical analyses), and the potential integration of information of large width census areas; the latter feature is essential when comparing the abundance of target fish species in areas with different disturbance levels (e.g. MPAs and areas open to fishing) due to the different approach distances to divers (or wariness) of commercial species associated with human disturbance [42, 44, 45]. The drawbacks of the DS method in comparison with fixed width standard UVC were: (1) the higher post-processing requirements to calculate parameters in comparison with CST, which might not be justified when other methods (e.g. FW3 or FW10) are accurate [45], (2) the need for at least 30–50 observations to apply the DS density calculations, which is a serious constraint at the species level for most commercial species outside marine reserves, and (3) the need to test or train the observers’ ability to accurately and precisely estimate distances. Regarding the second shortcoming, it can be overcome by using the average distance of each species to the transect as a way to calculate densities, which has been shown to be a robust estimator [35, 56]. However, it is important to note that it is difficult to produce accurate density estimates for rare and/or difficult to detect species with any UVC method [27, 35, 42]. For its part, density estimates may be seriously biased if distance estimates are wrong, so as with other UVC methods, initial training and an accuracy test among observers are recommended both to apply the DS method as well as for counts in narrow fixed width transects (FW3 and FW10). Considerable non-systematic variation in the ability of different observers to estimate distance underwater has been found in previous studies [15]. Biases that are not systematic either through space, time, method or observer can lead to misleading conclusions [14].

## Conclusions

Results of this study are relevant for the design of monitoring programs where the level of sampling effort would depend on a trade-off between the desired precision and logistic and material constraints. It is noteworthy that this method can be used for surveying other groups of marine animals (such as giant clams and mussels, sea turtles, lobsters, sharks, and rays) or objectives (e.g. locating fish nests, surveying caves used as refuges for fish and other groups, assessing spawning aggregations, etc.) [38, 52] or furthermore used to geo-locate survey sites (e.g. point counts). In addition, the spatial scale (width and length covered with TRT+DS) should be adjusted depending on environmental, ecological and logistic issues specific to each study. Specifically, we encourage the use of the TRT+DS as a rapid assessment method [57] for fish surveys in new sites not previously explored, in which divers could gather spatial ecological and ecosystem information on large areas while counting fishes.
